# The experience of northern italy in elderly patients with COVID-19 infection and mental disorders

**DOI:** 10.1192/j.eurpsy.2021.698

**Published:** 2021-08-13

**Authors:** G. Serafini, E. Bondi, C. Locatelli, M. Amore

**Affiliations:** 1 Department Of Neuroscience, Rehabilitation, Ophthalmology, Genetics, Maternal And Child Health (dinogmi), University of Genoa, IRCCS Ospedale Policlinico San Martino, Genoa, Italy, Genoa, Italy; 2 Department Of Psychiatry, Hospital Papa Giovanni XXIII, Bergamo, Italy, Bergamo, Italy; 3 Department Of Neuroscience, Rehabilitation, Ophthalmology, Genetics, Maternal And Child Health (dinogmi), Departimento di Neuroscienze, Università di Genova, Genoa, Italy

**Keywords:** COVID-19 infection, mental health, delirium, Psychological Distress

## Abstract

**Introduction:**

In December 2019, the first cases of Corona Virus Disease 2019 (COVID-19) outbreak related to acute respiratory syndrome coronavirus 2 (SARS-CoV-2) infection were reported in the Chinese city of Wuhan. European countries experienced a tragic growth in the number of Covid-19 cases although several restrictions have been imposed.

**Objectives:**

The study is aimed to describe the first experience of the Hospital Papa Giovanni XXIII in the city of Bergamo, Northern Italy.

**Methods:**

The most relevant clinical characteristics of aged patients with COVID-19 and mental disorders have been described.

**Results:**

According to the experience of the Hospital Papa Giovanni XXIII, medical departments, after appropriate training of all healthcare workers, have been rapidly converted into specific units aimed at treating patients with COVID-19 infection. Specifically, we directly observed a rapidly growing request of psychiatric interventions in aged patients with COVID-19 infection due to the emergence of severe delirium (mainly hyperkinetic) which was reported in approximately 30−50% of cases increasing with age, psychomotor agitation, anxiety, and depressive symptoms. When compared with younger subjects, we found that subjects aged 65 or above with prolonged hospitalization in our hospital are more vulnerable to: 1) environmental factors (e.g., social isolation and distance from family members, stay in intensive/subintensive units, communication difficulties due to therapeutic devices); 2) individual factors (e.g., COVID-19 possible neurotropic properties, impairments in insight and cognitive dysfunctions, comorbid medical conditions, and use of multiple medications).

**Conclusions:**

The main implications of the present findings have been discussed.

